# Distinct damage levels and transcriptional responses of lung in Hezuo pigs and Bama pigs during cold exposure

**DOI:** 10.5713/ab.250933

**Published:** 2026-03-11

**Authors:** Yajuan Li, Xiaoli Gao, Yating Zhang, Shuangbao Gun

**Affiliations:** 1 College of Animal Science and Technology, Gansu Agricultural University, Lanzhou, China; 2 Gansu Innovation Center for Swine Production Engineering and Technology, Lanzhou, China; 3 Gansu Diebu Juema Pig Science and Technology Backyard, Diebu, China

**Keywords:** Bama Pigs, Cold Exposure, Hezuo Pigs, Lungs

## Abstract

**Objective:**

The aim of the present study is to compare cold adaptation mechanisms between cold-tolerant Hezuo and cold-sensitive Bama pigs.

**Methods:**

A total of 40 healthy pigs (75 days old), including 20 Hezuo pigs and 20 Bama pigs, were used in this study. A 2×2 factorial design was employed, with factors including breed (Hezuo vs. Bama) and ambient temperature (23±2°C vs. −15±2°C). After 7 days of acclimation, pigs in the cold groups were exposed to low temperature and slaughtered on days 0, 1, 5, and 10 (n = 5 per group per time point). All pigs had ad libitum access to feed and water. Lung histology, wet/dry ratio, oxidative and inflammatory biomarkers, apoptosis, and transcriptomics were analyzed.

**Results:**

The results showed that Hezuo pigs displayed less severe alveolar septal thickening, inflammatory infiltration, and fine bronchial fold extension during cold exposure than Bama pigs. The W/D ratio dramatically decreased in Hezuo pigs while rising in Bama pigs. Hezuo pigs exhibited significantly higher aquaporin-1 (*AQP-1*) and aquaporin-5 (*AQP-5*) expressions than Bama pigs during the middle and late phases. Bama pigs displayed increased reactive oxygen species, malondialdehyde, tumor necrosis factor-alpha (*TNF-α*), interleukin-1 beta (*IL-1β*) and decreased glutathione (GSH) levels. Hezuo pigs maintained stable GSH levels and no significant changes in late-phase inflammatory markers. Bama pigs had a greater apoptosis density and more TUNEL-positive cells than Hezuo pigs, which was related to the down-regulation of B-cell lymphoma 2 (*Bcl-2*) and the up-regulation of Bcl2-associated X protein (*Bax*) and *Caspase-3*. Transcriptomic analysis revealed that in Bama pigs, distinctive genes such as mucin 5B (MUC5B), matrix metallopeptidase 9 (*MMP9*), alveolar macrophage chemotactic factor-II (*AMCF-II*), interleukin 22 receptor subunit alpha 1 (*IL22RA1*), C-C motif chemokine ligand 16 (*CCL16*), SRY-box transcription factor 9 (*SOX9*), keratin 5 (*KRT5*) contribute to mucus hypersecretion, extracellular matrix degradation, and sustained inflammatory chemotaxis, worsening tissue damage. In contrast, Hezuo pigs possess unique genes such as aldehyde dehydrogenase 1 family member A2 (*ALDH1A2*), acyl-CoA synthetase long chain family member 6 (*ACSL6*), *ACSM5*, aldo-keto reductase family 1 member C1 (*AKR1C1*), nuclear receptor subfamily 4 group A member 3/2 (*NR4A3/2*), G protein subunit gamma 4 (*GNG4*), glycogen synthase 2 (*GYS2*), which enhance lipid metabolism, facilitate aldehyde detoxification, and mitigate oxidative stress, thereby orchestrating a cellular protection.

**Conclusion:**

Hezuo pigs exhibit protective molecular mechanisms, suggesting potential targets for cold-resistance breeding.

## INTRODUCTION

Cold is a common source of stress in animal husbandry in northern China. It can have a series of negative effects on animals, which lead to the decrease of animal body temperature, loss of appetite, slow growth rate and decrease of immune function. Cold also increase the morbidity and mortality of a number of respiratory system diseases, which can cause significant financial losses for the swine industry [1]. The most important organ in the respiratory system is the lungs, which contain hundreds of millions of pulmonary alveolar. Because of the pulmonary alveolar’s rich capillary network and direct contact with cold air, when cold air enters the pulmonary alveolar, the temperature loss is greater than in other areas of the body [2].

Oxidative stress triggered by cold exposure can lead to complex pathological changes in lung tissue, including apoptosis, necrosis, enhanced inflammatory responses, and alveolar structural damage [3]. Apoptosis, a key feature of acute lung injury, is regulated by Caspase family proteins and influenced by the balance between pro-apoptotic Bcl2-associated X protein (BAX) and anti-apoptotic B-cell lymphoma 2 (Bcl-2) [4]. Cold stress has been shown to promote apoptosis by up-regulating *BAX* and down-regulating *Bcl-2* [5]. Altered expression of aquaporin 1 (AQP1) and aquaporin 5 (AQP5) has been linked to lung injury, edema, and other pathologies [6]. Furthermore, Cold-induced upregulation of pro-inflammatory cytokines such as tumor necrosis factor-alpha (*TNF-α*) and interleukin-1 beta (*IL-1β*) can further exacerbate local inflammatory and tissue damage, ultimately compromising animal health [7]. For example, studies such as Luo et al [8] indicate that cold exposure significantly exacerbates inflammatory responses in mouse lungs, characterized by substantial infiltration of neutrophils and macrophages into the alveolar spaces and marked upregulation of inflammatory mediators including interleukin 6 (*IL-6*), *TNF-α*, and *IL-1β*. While murine models provide valuable insights into cold stress physiology, their applicability to pigs remains limited. Distinct genetic backgrounds may lead to divergent cold response mechanisms among different pig breeds. For example, Jiao et al [9] discovered, based on liver transcriptome analysis, that two sheep breeds responded to cold exposure with distinct heat production mechanism. Altay lambs increased their cold tolerance by activating the liver-mediated muscle shivering/non-shivering heat production pathway, while the livers of Hu lambs did not participate in this process. *Peromyscus maniculatus* uses a temperature-dependent mild hypothermia strategy to maximize energy allocation in the frigid highland environment, while *Peromyscus leucopus* specifically increase its basal metabolic rate in response to prolonged cold exposure, according to a study by Hayward et al [10].

More fundamentally, compared to metabolic organs such as the liver and skeletal muscle, the lungs—as the body’s sole organ directly exchanging gases with the external environment—exhibit distinct patterns of cold-induced injury and molecular responses. Injuries to the liver and skeletal muscle primarily stem from cold-induced systemic energy deficit, insulin resistance, and oxidative stress. In contrast, the lungs bear the brunt of exposure to the physical irritation of dry cold air, heightened susceptibility to pathogens, and intense local immune inflammatory storms [11].

The cold-sensitive Bama pig and the cold-tolerant Hezuo pig, two local breeds with very different ecological origins, were chosen as models in this study to shed light on the phenotypic variations in cold adaptation among pig breeds. Due to its lengthy history of living in a subtropical humid climate zone with an average yearly temperature of 18°C–22°C, the Bama pig is comparatively poorly adapted to extremely cold temperatures due to a lack of selection pressure from prolonged low-temperature stress [12]. Hezuo pigs, on the other hand, have long lived in a cold, hypoxic, high-altitude environment at 2,800–4,200 meters above sea level, where the average winter temperature is only −8°C to 2°C. It possesses a strong resistance phenotype to cold and hypoxia as a result of long-term adaptive evolution under such circumstances [13]. By contrasting the physiological characteristics and transcriptome responses of Hezuo pigs under cold exposure, we sought to examine inter-breed variations in lung injury caused by cold exposure as well as the molecular processes of immune modulation of lung injury. These results will provide important candidate molecules for breeding cold-resistant pigs.

## MATERIALS AND METHODS

### Experimental animals and study design

Twenty Hezuo pigs (Diebu County, Gannan, Gansu Province) and twenty Bama pigs (Bama Yao Autonomous County, Hechi, Guangxi Zhuang Autonomous Region) were chosen for the cold stimulation test in this investigation. The pigs were healthy, 75 days old, and similar body weights. Eight groups of five replicates each were used for the test, which was set up with two temperatures (room temperature: 23±2°C; low temperature: −15±2°C) and two pig breeds (Bama and Hezuo). Throughout the test period, the pigs were fed as usual. Lung tissue samples were taken after the lung organizations were killed at 0 days, 1 day, 5 days, and 10 days of cold exposure. The remaining lung tissues were dried in an oven at 65°C, while some samples were kept at −80°C. The other portion was cleaned with phosphate-buffered saline (PBS), fixed, and kept in a 4% paraformaldehyde solution.

### Hematoxylin and eosin staining

Following sectioning, the right lung was removed, and the top lobe was immersed in 4% paraformaldehyde for a whole night before being imbedded in the substance. Lungs from five pigs in each group were chosen at random for the histopathological investigation. From each group, one tissue segment was chosen. Following normal protocols, the slices were stained with hematoxylin and eosin (HE). Three randomly chosen regions, each with 200 fields, were chosen for each lung segment in order to use ImageJ to quantify the amount of inflammatory infiltration and the thickness of the alveolar septa.

### TUNEL staining

The right upper lobe was fixed overnight in 4% paraformaldehyde, then dehydrated sequentially through 150 g/L and 300 g/L sucrose until tissue sedimentation. After flash-freezing on dry ice, samples were OCT-embedded and sectioned at 8 μm (−20°C). Sections were fixed in 40 g/L paraformaldehyde (30 min), washed 2/10 min in PBS, permeabilized with 0.1 % Triton X-100, and was followed by three PBS washes. It was performed using 50 μL reaction mixture 5 μL TdT enzyme and 45 μL labeling solution incubated at 37°C in dark and humidified for 1 h, followed by three PBS washes were performed on the cells. Nuclei were counterstained with DAPI. Apoptotic cells (green fluorescence) were visualized by fluorescence microscopy.

### Enzyme-linked immunosorbent assay

Following the instructions for the corresponding indicators of the enzyme-linked immunosorbent assay (ELISA) kit (Jiangsu Kete Biotechnology), the supernatant of isolated pig lung tissue was used to detect the levels of reactive oxygen species (ROS), malondialdehyde (MDA), glutathione (GSH), TNF-α and IL-1β. The OD values at 450 nm of samples were measured by the Microplate Spectrophotometer, and the concentration of each sample was calculated by standard curve.

### Lung wet/dry weight ratio

Fresh right lung tissue from pig was taken, and the right upper lobe was isolated for weighing and labeled as lung wet weight. Subsequently, the lung tissue was dehydrated for 48 h in an oven at 65°C, and lung dry weight was measured. The lung wet/dry (W/D) ratio of each pig was computed [14].

### RNA sequencing

Lung tissue samples were collected for transcriptome sequencing following 5 days of cold exposure. Lung tissue samples were collected for transcriptome sequencing following 5 days of cold exposure, forming four groups based on pig breed and treatment conditions: Bama pig room temperature group (BC, n = 5), Bama pig cold-treated group (BT, n = 5), Hezuo pig room temperature group (HC, n = 5), and Hezuo pig cold-treated group (HT, n = 5). Bama pig room temperature group (BC, n = 5), Bama pig cold-treated group (BT, n = 5), Hezuo pig room temperature group (HC, n = 5), and Hezuo pig treated group (HT, n = 5) were the four groups that were created based on the pig breeds and treatment circumstances.

RNA-seq and library construction were performed by Shanghai Peseno Biotechnology. Poly(A)-enriched porcine mRNA was fragmented, reverse-transcribed with random primers, and converted into Illumina sequencing libraries using the NEBNext Ultra II Kit, followed by size selection (450 bp) and polymerase chain reaction (PCR) amplification. After sequencing (PE150) on an Illumina platform, raw reads were quality-filtered (fastp) and aligned (HISAT2) to the *Sus scrofa* 11.1 reference genome. Using the DESeq2 (v1.38.3) software, we conducted a differential analysis of gene expression between the comparison groups. A significant p-value of less than 0.05, an adjusted p-value (FDR) of less than 0.05, and an expression fold change of more than 2 (|log2FoldChange|>1) were prerequisites for screening differentially expressed genes (DEGs). To do enrichment analysis on the differential genes, Cluster Profiler (v4.6.0) software was utilized. The p-value was determined using the hypergeometric distribution approach (p<0.05) after the differential genes were enriched using the cluster Profiler (v4.6.0) program. To identify the primary biological roles of the differential genes, they were all examined for function enrichment using the Kyoto Encyclopedia of Genes and Genomes (KEGG) and Gene Ontology (GO). The PPI is based on transcriptome sequencing, which first predicts interactions using the STRING database (https://cn.string-db.org/) and then uses Cytoscape v3.10.3 software to create a map of protein interactions.

### Reverse transcription quantitative polymerase chain reaction

Using the TransZol Up reagent, total RNA was extracted from around 0.1 g of lung organization. The extracted RNA was analyzed spectrophotometrically for purity (A260/A280 = 1.8–2.0) and concentration (800–1,000 μg/mL). Using the Prime Script reagent kit with gDNA Eraser, genomic DNA was extracted (42°C, 2 min) and cDNA was synthesized (37°C, 15 min). Suzhou Jinwei Zhi Biotechnology. generated the primers based on the NCBI porcine gene sequences (Table 1 for primer sequences). The quantitative polymerase chain reaction (qPCR) reaction system (20 μL total volume) consisted of: 10 μL SYBR Green Pro Taq HS qPCR Mix, 0.8 μL each of forward and reverse primers (10 μM), 2 μL cDNA template, and 6.4 μL RNase-free water. The amplification protocol included: initial denaturation at 94°C for 2 min; 40 cycles of 94°C for 15 s, 60°C for 15 s, and 72°C for 10 s; followed by a final extension at 72°C for 10 min. Melt curve analysis was performed to confirm amplification specificity. All reactions were run in triplicate Using *GAPDH* as the internal reference gene, the 2-ΔΔCt method was used to compute the relative table of target genes.

### Statistical analysis

Statistical analysis was performed using IBM SPSS Statistics ver. 26.0 (IBM) and GraphPad Prism ver. 8.3.0.538 (GraphPad Software). To determine the significance of differences between two groups, the two-tailed unpaired Student’s t-test was employed. For analyzing the significance of differences among multiple groups, one-way analysis of variance (ANOVA) with Duncan’s multiple range test was employed. Different letters indicate significant differences among treatment groups within the same pig breed (^A–C^ p<0.01; ^a–c^ p<0.05). Asterisks indicate significant differences between pig breeds under the same treatment conditions (* p<0.05; ** p<0.01). The same applies below. All data were presented as mean±standard deviation (SD).

## RESULTS

### Lung histology changes after cold exposure to Hezuo and Bama pigs

**Figure 1:**
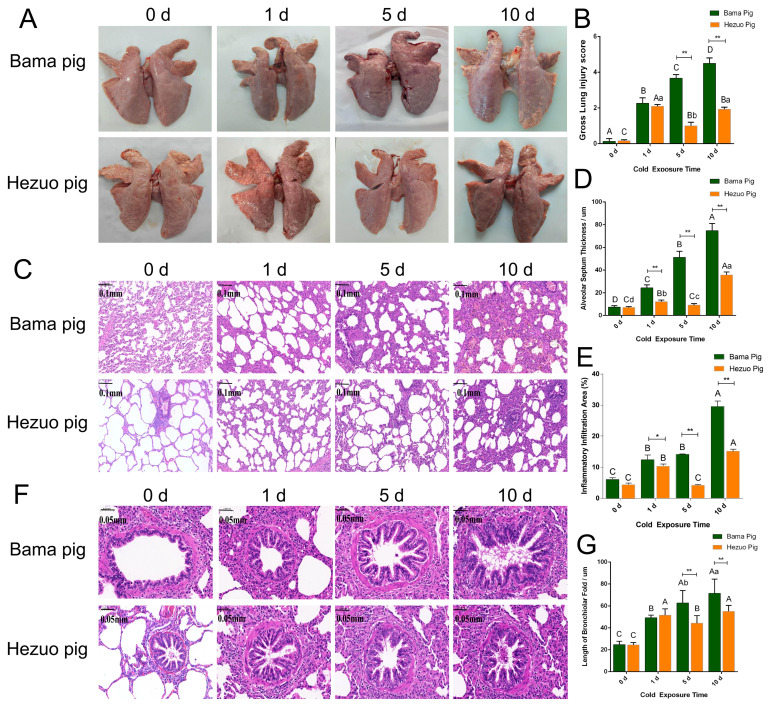
Lung organization pathology results. (A) Appearance and morphology of pig lungs. (B) Gross lung injury score chart. (C) HE stains results (alveolar 200×). (D) Histogram of alveolar septal thickness analysis. (E) Inflammatory infiltration area. (F) HE stains results (bronchial 400×). (G) Histogram of bronchial fold length analysis. Different letters indicate significant intra-breed differences among treatment groups over time (^A–D^ p<0.01; ^a–d^ p<0.05). Asterisks indicate significant inter-breed differences at the same treatment time point (* p<0.05; ** p<0.01). HE, hematoxylin and eosin.

From the observation of the appearance of pigs, it was found that the lung color became dark and edema after cold exposure (Figure 1A). The damage score confirmed that Bama pigs were more severely damaged at each time point than Hezuo pigs, and there was a significant difference between the breeds at 5 d and 10 d (p<0.01) (Figure 1B). HE results showed that cold exposure caused structural damage to the lung tissues of Bama pigs and Hezuo pigs, and there were significant differences in the degree of damage. In the control group, both varieties showed normal lung structure, complete alveolar septum, and no obvious pathological condition. After cold exposure, alveolar septal rupture, thickening, edema, decreased number of alveoli per unit area, infiltration of inflammatory cells and prolonged longitudinal plica of bronchioles occurred in both breeds, and the degree of injury in Bama pigs was significantly higher than that in Hezuo pigs. Statistics showed that after cold exposure, the alveolar septum thickness and bronchiole wrinkle length of Hezuo pigs and Bama pigs increased significantly. During the 5 days of cold exposure, there were significant differences in alveolar septum thickness and bronchiole wrinkle length between the two breeds, and the alveolar septum thickness and bronchiole wrinkle length of Hezuo pigs at this time were significantly lower than those of cold exposure for 1 day, but this did not occur in Bama pigs (Figures 1C–1G). This result is consistent with the findings of the gross pathological observations.

### The effects of cold exposure on water metabolism-related indices in the lungs of Hezuo and Bama pigs

**Figure 2:**
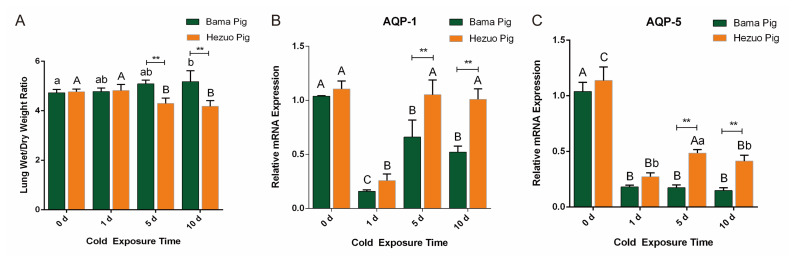
Results of water metabolism-related indexes in lungs. (A) W/D ratio. (B) *AQP-1* mRNA level in lungs. (C) *AQP-5* mRNA level in lungs. Different letters indicate significant intra-breed differences among treatment groups over time (^A–C^ p<0.01; ^a,b^ p<0.05). Asterisks indicate significant inter-breed differences at the same treatment time point (** p<0.01). *AQP-1*, aquaporin-1; *AQP-5*, aquaporin-5; W/D, wet/dry.

In comparison to the control group, the W/D ratio of Bama pigs rose in a time-dependent way after cold exposure, particularly at 5 and 10 days (p<0.05). However, the W/D ratio of Hezuo pigs remained stable on the first day of cold exposure and decreased sharply on the fifth and tenth days (p<0.01) (Figure 2A). Compared with the control group, the levels of *AQP-1* and *AQP-5* were decreased in the cold exposure group, and there was a significant difference (p<0.01) between the two varieties on the fifth day of cold exposure (Figures 2B, 2C).

### Impact of cold exposure on Hezuo and Bama pigs’ lung apoptosis levels

**Figure 3:**
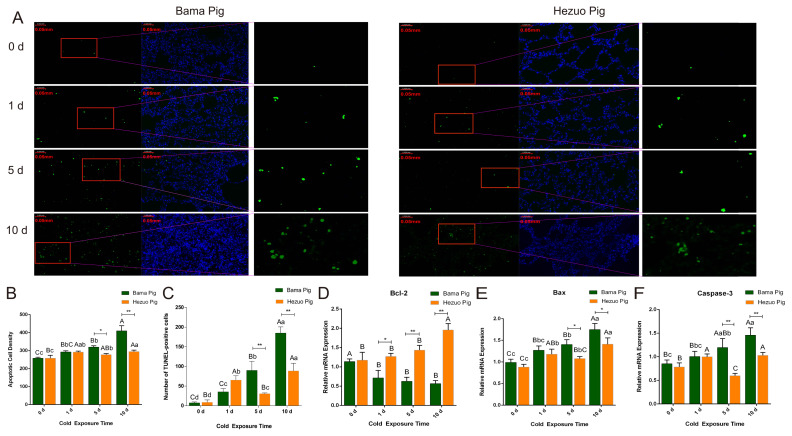
Results of apoptosis analysis in lungs. (A) TUNEL staining results (green fluorescence indicates apoptotic cells, blue fluorescence indicates normal nuclei, 400×). (B) Apoptosis optical density analysis bar chart. (C) Number of TUNEL-positive cells. (D) Level of *Bcl-2* in lungs. (E) Level of *Bax* in lungs. (F) Level of *Caspase-3* in lungs. Different letters indicate significant intra-breed differences among treatment groups over time (^A–C^ p<0.01; ^a–d^ p<0.05). Asterisks indicate significant inter-breed differences at the same treatment time point (* p<0.05; ** p<0.01). *Bcl-2*, B-cell lymphoma 2; *Bax*, Bcl2-associated X protein.

The TUNEL staining and quantitative analysis results indicated that cold exposure induced apoptosis in the lung tissues of both pig breeds, with significant differences observed between the breeds (Figure 3A). In terms of apoptosis density, although both breeds showed an overall increasing trend after cold treatment, Bama pigs consistently exhibited significantly higher apoptosis density than Hezuo pigs. Specifically, after 1 day of cold treatment, the apoptosis density of both breeds increased significantly (p<0.05), but no significant difference was observed between the two groups. By 5 days of cold treatment, the apoptosis density of Bama pigs continued to rise, while Hezuo pigs showed a slight decline, resulting in a significant difference between the two breeds (p<0.05). At 10 days of cold treatment, the apoptosis density of both breeds increased further, with Bama pigs still displaying significantly higher (p<0.01) apoptosis density than Hezuo pigs (Figure 3B). Statistical results for TUNEL-positive cell counts aligned with these trends: Bama pigs exhibited a continuous increase in positive cells over time during cold treatment; Hezuo pigs showed a significant rise at day 1, a significant decline at day 5 (when significant differences emerged between breeds), and a renewed significant increase (p<0.01) by day 10 (Figure 3C). According to the results of apoptosis-related gene expression, *Bcl-2* expression dramatically increased (p<0.01) in Hezuo pigs following cold exposure, while it significantly decreased (p<0.01) in Bama pigs. In Bama pigs exposed to extended cold exposure, there was a significant increase (p<0.05) in the expression of *Bax* and *Caspase-3*. On the other hand, Hezuo pigs’ expression of *Bax* and *Caspase-3* varied in an “increase-decrease-increase” pattern (Figures 3D–3F).

### The effect of cold exposure on oxidative stress and the inflammatory response in the lung tissue of Hezuo and Bama pigs

**Figure 4:**
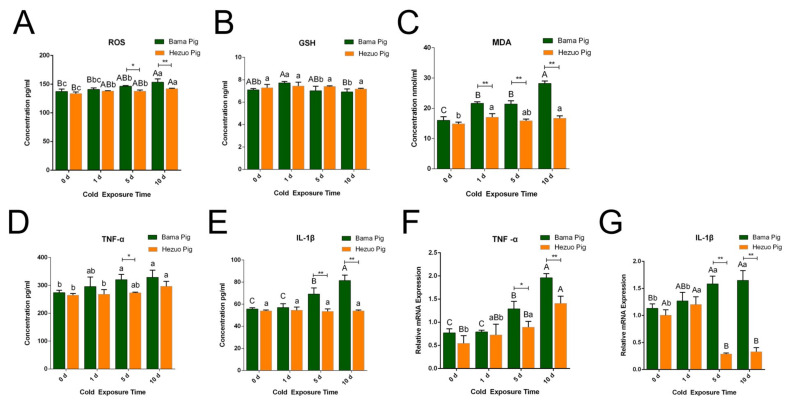
Results of oxidative stress and inflammatory response in lungs. (A–C) ELISA was used to detect the dynamics of ROS, GSH, and MDA protein concentration. (D, E) ELISA to detect the dynamics of TNF-α and IL-1β protein concentrations. (F,G) qPCR to detect relative *TNF-α* and *IL-1β* mRNA expression. The units of each index in panels (A–E) are the standard detection units recommended by the corresponding ELISA kits. Different letters indicate significant intra-breed differences among treatment groups over time (^A–C^ p<0.01; ^a–c^ p<0.05). Asterisks indicate significant inter-breed differences at the same treatment time point (* p<0.05; ** p<0.01). ROS, reactive oxygen species; GSH, glutathione; MDA, malondialdehyde; TNF-α, tumor necrosis factor-alpha; IL-1β, interleukin-1 beta; ELISA, enzyme-linked immunosorbent assay; qPCR, quantitative polymerase chain reaction.

According to the ELISA results, Bama pigs reacted more strongly to cold exposure, and as the duration of cold exposure increased, ROS and MDA concentrations tended to rise while GSH concentrations tended to fall. After one day of cold exposure, Bama pigs’ ROS (p<0.05) and MDA (p<0.01) concentrations significantly increased; after five days of cold exposure, the ROS and GSH concentrations of Bama pigs continued to rise significantly (p<0.05) and fall significantly (p<0.05) respectively; and after ten days of cold exposure, the concentrations of ROS, GSH, and MDA of Bama pigs peaked. In contrast, at 5 days of cold exposure, the Hezuo pigs showed no significant change in GSH, a smaller increase in ROS/MDA and an intermediate regulatory node, and a significant decrease in MDA (p<0.05); at 10 days of cold exposure, ROS/MDA increased, but it was significantly lower (p<0.01) than that in the Bama pigs (Figures 4A–4C). Breed-specific inflammatory responses were also observed. In Bama pigs, TNF-α and IL-1β protein concentrations increased steadily, according to ELISA data; in Hezuo pigs, only TNF-α increased slowly (lower than in Bama pigs, p<0.05), and IL-1β did not change substantially. *TNF-α* mRNA increased significantly in both breeds (p<0.01) and peaked 10 days after cold exposure, according to qPCR data; nevertheless, Hezuo pigs’ expression was significantly lower than that of Bama pigs. However, Hezuo pigs consistently showed lower expression than Bama pigs, and starting on day 5, the difference became significant (p<0.05). A differentiation pattern in *IL-1β* gene expression was observed, with a substantial rise over time in Bama pigs (p<0.05). Significant inhibition occurred in the middle phase (5 days of cold exposure, p<0.01), an increase occurred in the acute phase (1 day of cold exposure, p<0.05), and stability occurred in the late phase (10 days of cold exposure) in Hezuo pigs (Figures 4D–4G).

### Analysis of differentially expressed genes in the lungs of Hezuo pigs and Bama pigs under cold exposure

Based on the significant cold tolerance differences between Hezuo and Bama pigs, previous studies identified the 5-day cold exposure as a critical phenotypic divergence point. At this stage, Hezuo pigs exhibited an active alleviation of lung tissue damage, along with significantly lower levels of oxidative stress (e.g., ROS, MDA), pro-inflammatory cytokines (e.g., TNF-α, IL-1β), and apoptosis compared to Bama pigs, suggesting the activation of effective anti-injury mechanisms. However, the global transcriptional regulatory network underlying these phenotypic differences remains unclear. Therefore, this study focuses on the 5-day cold exposure time point and employs RNA-seq to systematically compare the transcriptomic profiles of lung tissues between the two breeds. The aim is to identify DEGs and perform GO and KEGG enrichment analyses, thereby revealing key pathways and regulatory genes associated with cold tolerance in Hezuo pigs and providing theoretical insights into their anti-cold stress mechanisms.

**Figure 5:**
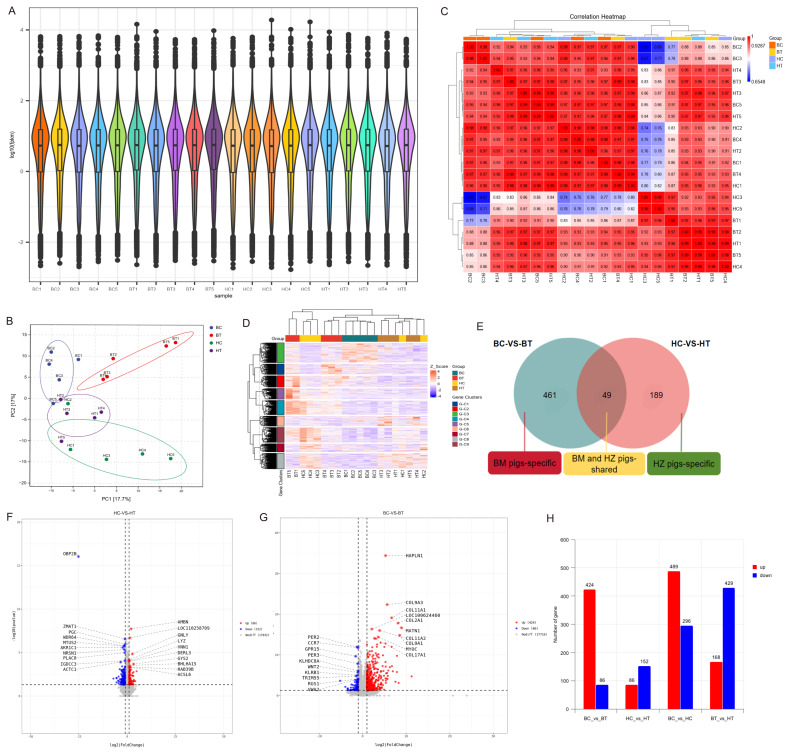
Transcriptome analysis of cold exposure lungs in two pig breeds. (A) Violin plot. (B) 2D PCA plot. (C) Correlation analysis plot. (D) Cluster analysis of DEGs. (E) Venn. (F, G) Volcano plot. (H) Differential expression results statistics. BC, Bama pig room temperature group; BT, Bama pig cold-treated group; HC, Hezuo pig room temperature group; HT, Hezuo pig treated group; DEGs, differentially expressed genes.

To further investigate the molecular characteristics of the cold adaptation of porcine lungs from two pig breeds, we performed RNA sequencing (RNA-seq) analysis on lung samples from 20 pigs. All samples had consistent and dependable RNA-seq data quality, with GC content (>47.55%), Q20 (>98.28%), Q30 (>95.04%), clean reads (>98.29%), and clean data (>98.01%) satisfying the requirements (Supplement 1). According to violin plot results, there were no discernible individual differences in the sequencing data of the 20 test samples, which were uniformly distributed (Figure 5A). At the transcriptome level, the four groups (BC, BT, HC, and HT) were significantly separated by PCA and inter-sample correlation analysis, with good intra-group repeatability (Figures 5B, 5C). The samples’ clustering into the four groups and notable differences were shown by cluster analysis of DEGs (Figure 5D). A total of 510 DEGs, comprising 424 up-regulated and 86 down-regulated genes, were found in the Bama pig group (BT vs BC) using the criteria of |log2FoldChange|>1, p<0.05 and adjusted p-values (FDR)<0.05. Only 238 DEGs, comprising 86 up-regulated and 152 down-regulated genes, were found in the Hezuo pig group (HT vs HC) (Figure 5H). Interestingly, the percentage of up-regulated genes in Bama pigs was 83.1% (424 out of 510), which significantly higher than the percentage of that were down-regulated genes, which was 16.9% (86 out of 510). In Hezuo pigs, the ratio of genes that were up-regulated and down-regulated was around 1:1.8 (Figures 5F, 5G). Only 49 DEGs were shared by the two breeds, according to a Venn diagram study (9.6% of Bama pig DEGs and 20.6% of Hezuo pig DEGs). For Hezuo pigs, the ratio of DEGs specific to Bama pigs was as high as 461 (90.4%) and 189 (79.4%) (Figure 5E).

### Pathway enrichment analysis and functional annotation of Hezuo pig-specific genes under cold exposure

**Figure 6:**
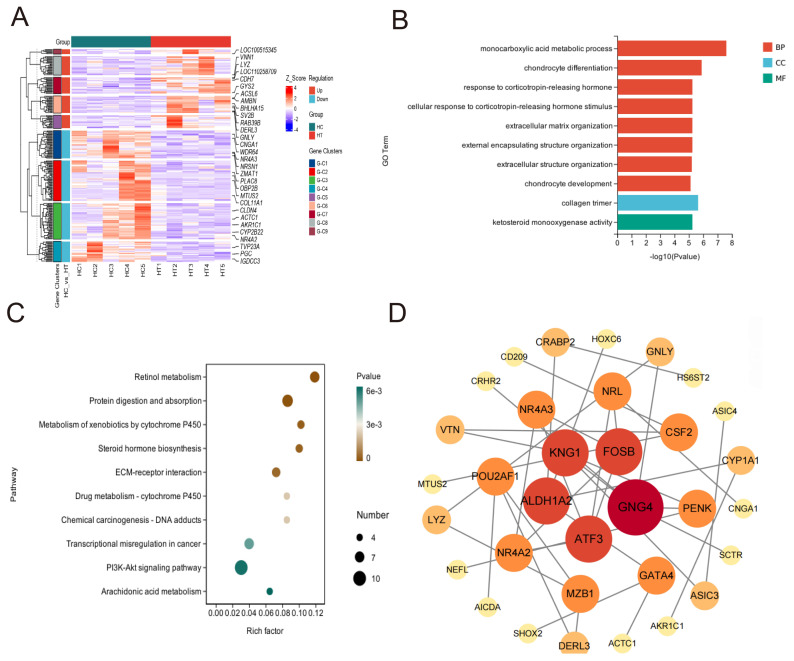
Enrichment analysis of differentially expressed genes specific to Hezuo pigs. (A) Heatmap of DEGs in HC vs HT. (B) GO function annotation of DEGs in HC vs HT group. Vertical coordinates are GO Term. (C) KEGG pathway enrichment analysis of DEGs in the HC vs HT group. Vertical coordinate is KEGG pathway. (D) Protein network interactions analysis of specific DEGs of HC vs HT. HC, Hezuo pig room temperature group; HT, Hezuo pig treated group; DEGs, differentially expressed genes; GO, Gene Ontology; KEGG, Kyoto Encyclopedia of Genes and Genomes.

**Figure 7:**
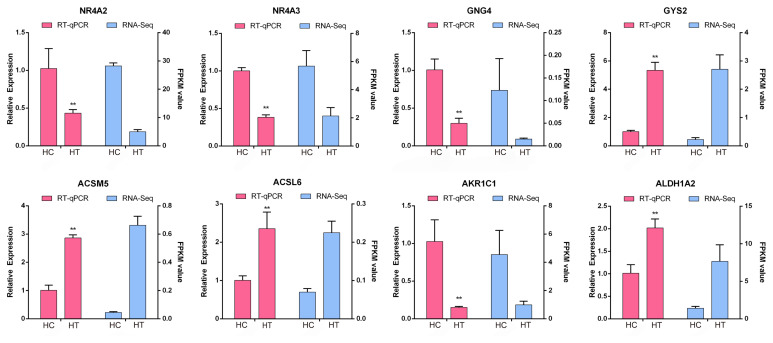
Comparison of RNA-Seq and RT-qPCR results of Hezuo pig-specific DEGs. RT-qPCR, reverse transcription quantitative polymerase chain reaction; HC, Hezuo pig room temperature group; HT, Hezuo pig treated group; DEGs, differentially expressed genes.

Transcriptome sequencing of Hezuo pig lung organizations under cold exposure circumstances revealed 189 distinct DEGs, comprising 76 up-regulated genes and 113 down-regulated genes (Figure 5E). We discovered that the significantly enriched biological processes were primarily engaged in important biological pathways such as metabolic regulation, inflammation, and oxidative stress by examining the GO enrichment of DEGs in the HC vs HT group (Figure 6B). The cellular response to corticotropin-releasing hormone stimulus and response to corticotropin-releasing hormone entries showed significant enrichment in terms of inflammation and oxidative stress; similarly, the metabolic process of monocarboxylic acid metabolic process and the ketosteroid monooxygenase activity entries showed significant enrichment in terms of metabolism. Aldehyde Dehydrogenase 1 Family Member A2 (*ALDH1A2*) was highly enriched in oxidative stress, Acyl-CoA Synthetase Long Chain Family Member 6 (*ACSL6*) and Acyl-CoA Synthetase Medium Chain Family Member 5 (*ACSM5*) in energy metabolism, and Aldo-Keto Reductase Family 1 Member C1 (*AKR1C1*), Nuclear Receptor Subfamily 4 Group A Member 3/2 (*NR4A3/2*) in the regulation of inflammatory factors (Supplement 2). There were significantly more genes involved in cold adaptation in the metabolic and environmental information processing pathways of Hezuo pigs, according to KEGG pathway analysis. The PI3K-Akt signaling pathway, a central metabolic regulatory hub, emerged as a pivotal signaling pathway in Hezuo pigs’ response to cold temperatures by integrating energy supply and stress response (Figure 6C). Glycogen Synthase 2 (*GYS2*) is enriched in this pathway (Supplement 3). The PI3K-Akt signaling pathway is located at the core of this pathway and is closely linked to numerous others. PPI analysis showed that the DEGs unique to Hezuo pigs formed a regulatory network with an average node degree of 0.719. 50 interaction edges and 139 nodes made up this network. The genes that occupied core locations were *ALDH1A2*, and G Protein Subunit Gamma 4 (*GNG4*) (Figure 6D). The above results identified the specific core candidate genes (*ALDH1A2*, *ACSL6*, *ACSM5*, *AKR1C1*, *NR4A3*, *NR4A2*, *GNG4*, and *GYS2*) in response to cold exposure in Hezuo pigs. The analysis of the expression of these 8 unique genes was done, and the results showed that the reverse transcription quantitative polymerase chain reaction (RT-qPCR) results followed the same trend as the RNA-Seq results (Figure 7).

### Pathway enrichment analysis and functional annotation of Bama pig-specific genes under cold exposure

**Figure 8:**
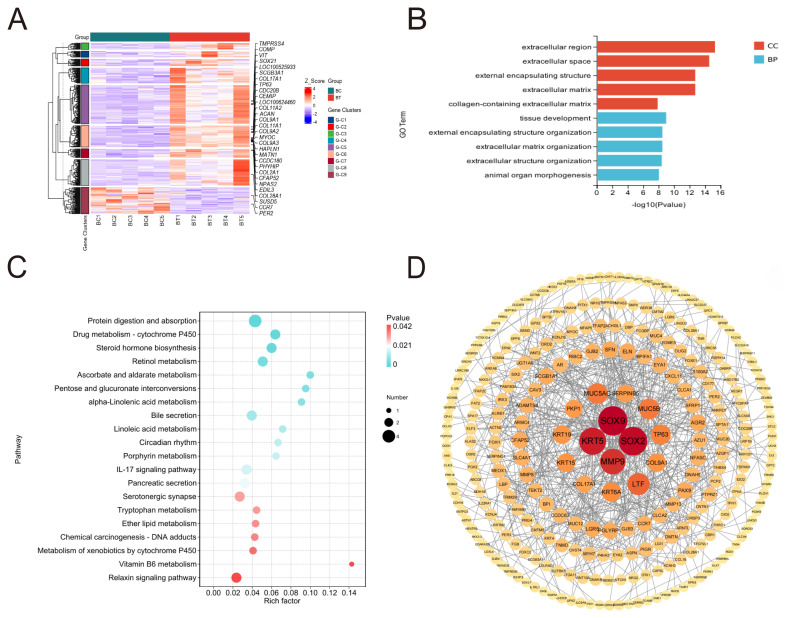
Enrichment analysis of differentially expressed genes specific to Bama pigs. (A) Heatmap of DEGs of BC vs BT. (B) GO functional annotation of unique DEGs of BC vs BT. (C) Protein network interactions analysis of unique DEGs of BC vs BT. (D) KEGG pathway enrichment analysis of unique DEGs of BC vs BT. BC, Bama pig room temperature group; BT, Bama pig cold-treated group; DEGs, differentially expressed genes; GO, Gene Ontology; KEGG, Kyoto Encyclopedia of Genes and Genomes.

**Figure 9:**
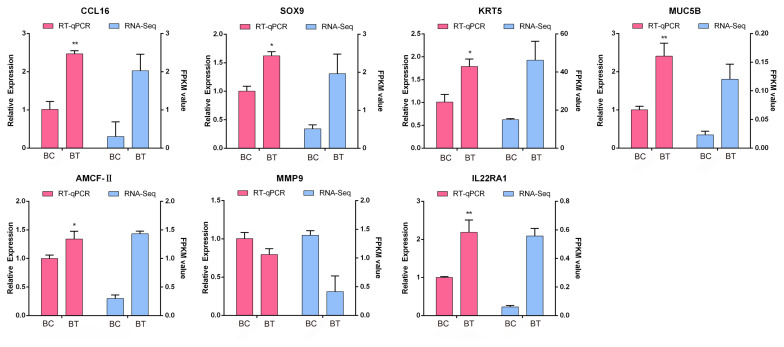
Comparison of RNA-seq and RT-qPCR results of Bama pig-specific DEGs. * p<0.05; ** p<0.01. BC, Bama pig room temperature group; BT, Bama pig treated group; RT-qPCR, reverse transcription quantitative polymerase chain reaction; DEGs, differentially expressed genes.

Under the same cold exposure circumstances, 461 DEGs were found in the lungs of Bama pigs, 377 of which were considerably up-regulated and 84 of which were significantly down-regulated (Figure 5E). The DEGs were substantially enriched in biological processes such as extracellular matrix (ECM) remodeling, tissue development, and organ morphogenesis, according to the GO enrichment data (Figure 8B). Further KEGG pathway analysis showed that the lungs of Bama pigs reacted to cold exposure mostly through signaling pathways associated with inflammation and immunity. Interestingly, the greatest notable enrichment was found in the IL-17 signaling pathway (Figure 8D). Interestingly, the majority of the DGEs in this pathway were found in downstream effector molecules, such as the matrix metalloproteinases matrix metallopeptidase 9 (*MMP9*), the inflammatory regulator lveolar Macrophage chemotactic factor-II (*AMCF-II*), and the mucus secretion-related gene genes-mucin 5B (*MUC5B*). Conversely, the cytokine-cytokine receptor interaction pathway and associated pathways showed a high enrichment of genes such as *AMCF-II*, interleukin 22 receptor subunit alpha 1 (*IL22RA1*), and C-C motif chemokine ligand 16 (*CCL16*) (Supplement 4). The DEGs unique to Bama pigs established a regulatory network with an average node degree of 3.22, according to PPI analysis. There were 632 interacting edges and 392 nodes in this network. SRY-Box Transcription Factor 9 (*SOX9*), *MMP9*, *MUC5B*, Keratin 5 (*KRT5*), and other genes were shown to be occupying core locations (Figure 8C). The above results identified the specific core candidate genes (*MUC5B*, *MMP9*, *AMCF-II*, *IL22RA1*, *CCL16*, *SOX9*, and *KRT5*) in response to cold exposure in Bama pigs. Likewise, the quantitative analysis of the seven distinct DEGs of Bama pigs was also carried out, and the outcomes were consistent with the RNA-Seq expression trend, demonstrating the excellent reliability of the sequencing data (Figure 9).

## DISCUSSION

The temperature and duration of low temperature environment are the primary determinants of the pathophysiological alterations of lung injury brought on by cold stress as a stressor [15]. For example, Joo et al [16] further demonstrated that chronic cold exposure resulted in persistent inflammatory response in the lungs of mice, which was manifested by neutrophils infiltration and a significant up-regulation in the expression of pro-inflammatory factors such as IL-12 and IL-17. In this study, Hezuo pigs exhibited greater cold resistance, whereas Bama pigs developed progressive pulmonary edema, alveolar structural deterioration, and inflammatory infiltration. These observations reflect notable inter-breed variations, yet align with the general pattern of cold-induced lung injury. The differential regulation of water transport proteins, particularly the recovery of *AQP-1* and *AQP-5* expression in Hezuo pigs during later stages of cold exposure, may contribute to their enhanced resistance to pulmonary edema and maintained fluid balance. In contrast, Bama pigs showed greater susceptibility to water-metabolic disruption under prolonged cold stress. These findings suggest the existence of a breed-specific cold adaptation mechanism in domestic pigs, with Hezuo pigs possessing a distinct tolerance to cold-induced lung damage.

Liu et al [5] confirmed that cold exposure could induce a significant increase in MDA content in lung tissue of mice and accompanied by GSH depletion. Luo et al [8] further reported that low temperature exposure could significantly increase the levels of inflammatory factors (such as IL-6, TNF-α) in lung tissue of rats, which in turn mediated lung injury. Wei et al [17] also observed the synergistic effect of acute cold stress on the increase of MDA content in lung tissue. The lung tissues of Bama pigs in the current study exhibited a more severe oxidative stress response (a marked rise in ROS and MDA and a sharp drop in GSH) as well as a prolonged pro-inflammatory response. In the present study, Bama pigs exposed to cold conditions exhibited severe oxidative stress, marked by significant rises in ROS and MDA and a sharp decline in GSH, along with a sustained pro-inflammatory response featuring persistently high expression of TNF-α and IL-1β. In contrast, Hezuo pigs showed only minimal changes in these parameters. Notably, Hezuo pigs demonstrated superior maintenance of oxidative homeostasis and more effective control of inflammation compared to the prolonged high-stress state observed in Bama pigs. These findings highlight breed-specific differences in stress response mechanisms and suggest that Hezuo pigs possess enhanced antioxidant and anti-inflammatory capacities that mitigate cold-induced lung damage.

Apoptosis is a crucial mechanism of programmed cell death and a fundamental pathophysiological characteristic of cold exposure-induced lung injury [18,19]. For example, cold stimulation dramatically reduced the Bcl-2/Bax ratio and triggered the Caspase-3/9 pathway in the hearts of broiler chickens, as shown by Wei et al [20]. Liu et al [5] that long-term cold exposure increased the expression of apoptosis-associated proteins in the lungs of mice (there was a considerable down-regulation of Bcl-2 and an over expression of Bax and Caspase-3). The present study, Bama pigs exhibited persistent apoptotic dysregulation under cold exposure, marked by sustained upregulation of Bax and Caspase-3, significant suppression of Bcl-2, and progressively increasing apoptotic density. In contrast, Hezuo pigs initiated effective defensive responses after five days of cold exposure, showing significant Bcl-2 upregulation, reduced apoptotic density, and normalized expression of pro-apoptotic factors. But by the tenth day of exposure to cold, apoptosis levels had increased once more. This could be because adaptive capacity had been depleted and a secondary stress response had been triggered, leading to an increased density of apoptotic cells. The variations imply that the control of apoptosis in lung organization under cold exposure exhibits notable breed specificity.

When exposed to cold, Hezuo and Bama pigs display different lung transcriptome traits and adaptation processes. Significant interbreed variations in cold-induced lung pathological damage, oxidative stress levels, inflammatory responses, apoptosis, and aquaporin expressions have been shown by our earlier multi-time-point phenotypic assessments (one day, five days, and ten days of cold exposure). The peak of phenotypic divergence and a crucial turning point for Hezuo pigs to start cold adaptation was found to be five days of cold exposure among these time points. In particular, Hezuo pigs had already shown signs of repair after five days of cold exposure. There was less damage to the lungs (for example, the alveolar septum was thinner than it was after one day), more antioxidant power (stable GSH levels and lower MDA), less inflammation (lower *IL-1β* expression), and better protection against cell death (higher *Bcl-2*). On the other hand, after five days of cold exposure, Bama pigs’ lung injury continued to worsen. Significantly decreased ROS/MDA concentrations, steady GSH levels, and less severe lung damage in comparison to Bama pigs indicate that these adaptive characteristics of Hezuo pigs were further solidified at 10 days of cold exposure. Therefore, to systematically examine the variations in gene expression between the two breeds and their underlying regulatory mechanisms during cold adaptation, we chose five days of cold exposure as the crucial time point for transcriptome sequencing in the current work.

Transcriptome sequencing technology has emerged as a crucial instrument for examining the molecular processes underlying animals’ adaptation to low temperatures in recent years. Important biological mechanisms and signaling pathways linked to thermoregulation can be uncovered by this method [21]. Many species, including cattle [22], sheep [23], and mice [24], have been successfully studied under cold exposure using this technique, which offers a crucial foundation for comprehending the molecular underpinnings of animal environmental adaptability. In this study, Hezuo pigs had substantially fewer DEGs and enriched pathways under cold exposure than Bama pigs. This implies that Hezuo pigs have some degree of cold adaptation and are less impacted by cold exposure. This result is consistent with research by Li et al [25], which found that when exposed to cold, the skeletal muscles of Mashen pigs had fewer DEGs and richer pathways than those of Large White pigs.

The majority of the candidate genes found in Hezuo pigs exposed to cold, including *ALDH1A2*, *ACSL6*, *ACSM5*, *AKR1C1*, *NR4A2*/*NR4A3*, *GNG4*, and *GYS2*, are functionally linked to metabolic adaptation, anti-inflammatory control, and antioxidant defense. *GNG4* is the most densely linked core gene and is essential to the signaling cascade of G protein-coupled receptors (GPCRs). A regulatory cascade in which GNG4-mediated GPCR signaling may stimulate the expression of *NR4A2*/*NR4A3*, thus upregulating *ALDH1A2*, which is implicated in retinoic acid production, is suggested by its interactions with the antioxidant gene *ALDH1A2* and the putative transcription factors *NR4A2/NR4A3* [26]. By regulating lipid metabolism and anti-inflammatory reactions, this regulatory cascade may provide protection against cold exposure. It is closely aligned with the retinol metabolism route found in the enrichment analysis (Figure 6C). Subsequent investigation shows that the *GNG4*/*NR4A* signaling axis uses a multi-level regulatory mechanism to coordinately maintain metabolic balance. On the one hand, this route may improve detoxification through controlling the gene *AKR1C1*, which is in charge of eliminating aldehyde compounds produced during oxidative stress [27]. In contrast, it activates medium-chain fatty acids to meet energy demands [28], upregulates *GYS2*-activating long-chain fatty acids to support phospholipid synthesis and pulmonary structural integrity [29], and regulates glycogen synthesis to facilitate energy storage and mobilization [30]. Preventing lung damage brought on by the cold.

The putative genes *MUC5B*, *MMP9*, *AMCF-II*, *IL22RA1*, *CCL16*, *SOX9*, and *KRT5* were shown to respond uniquely to cold exposure in Bama pigs. These genes were all significantly enriched in GO keywords and KEGG pathways, which are mostly involved in processes including mucus secretion, oxidative stress, and the inflammatory response. Within the network, *SOX9* serves as a densely interconnected hub transcription factor. According to functional investigations, this gene has the ability to dramatically increase *MMP9* production, which efficiently propels the ECM remodeling process [31]. The substantial enrichment of ECM-related GO functional words found in Bama pig lung tissues (Figure 8B) is entirely compatible with this regulating mechanism. In the meantime, results showing that inflammatory stimuli like TNF-α regulate MUC protein expression are consistent with the *SOX9*-mediated elevation of *MUC5B* [32]. Subsequent investigation indicates that *SOX9* may also interact functionally with the epithelial structural protein *KRT5* and the pro-inflammatory and pro-fibrotic chemokine *CCL16*. As a chemokine with both pro-inflammatory and pro-fibrotic properties, *CCL16* can work in concert with inflammatory cytokines like TNF-α and IL-1β to worsen damage to the alveolar-capillary barrier and encourage lung fibrosis [33].

There are notable breed-specific differences in the lung damage responses of Bama and Hezuo pigs when exposed to cold, and these unique adaptive strategies point to a genetic basis for the variance in cold tolerance between pig breeds. This aligns with contemporary research highlighting the genetic and physiological plasticity of livestock in response to environmental challenges across species. Recent studies have shown that intermittent cold stimulation can induce cardiac metabolic adaptation in broilers [34], and that energy deficiency at high altitude triggers specific microRNA responses in yak liver [35]. These findings collectively underscore the importance of organism-level metabolic reprogramming as a key adaptive mechanism. In contrast to approaches using dietary additives like coated cysteamine to enhance cattle performance [36], our study underscores the potential of harnessing intrinsic genetic diversity for improving environmental resilience. Future research will concentrate on integrating genome-wide genetic variation data for in-depth analysis to methodically clarify the molecular mechanisms underlying the observed inter-breed disparities, with particular attention to conserved stress-response pathways identified in other species.

## CONCLUSION

**Figure 10:**
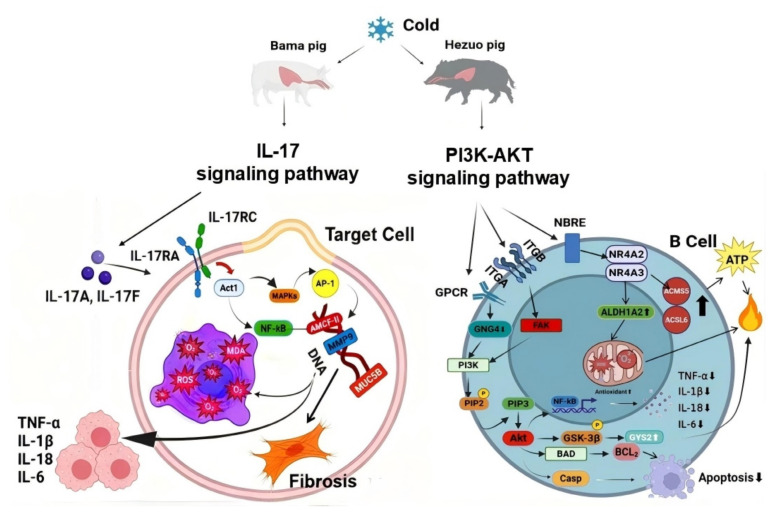
Differential response mechanisms in the lungs of Hezuo and Bama Pigs to cold exposure. Graphical abstract created with BioRender.com. IL, interleukin; TNF-α, tumor necrosis factor-alpha; GPCR, G protein-coupled receptor.

During cold exposure, Bama pigs were severely injured and exhibited pulmonary edema, oxidative stress, and inflammation, and apoptosis mediated by genes such as *MMP9*, *MUC5B*, *AMCF-II*, and *IL22RA1*, et al. which could serve as an early warning marker of cold sensitivity. In contrast, Hezuo pigs were highly resistant to cold with mild injury, and their protective mechanisms were the rapid regulation of water channel proteins to maintain fluid homeostasis, and the maintenance of oxidative homeostasis and anti-inflammatory effects, mediated by key genes such as *ALDH1A2*, *GYS2*, *ACSL6*, and *AKR1C1*, et al (Figure 10).

## Figures and Tables

**Table 1:** Primer sequence

Genes	Primer sequence (5’→3’)	Length	Number
*IL-1* *β*	F: CAAGAGGATACTTCCCCTGACC	150	NM_001302388.2
	R: CTTCCTTGGCAGGTTCAGGTA		
*TNF-* *α*	F: GCACTGAGAGCATGATCCG	161	NM_214022.1
	R: AACCTCGAAGTGCAGTAGG		
*Bcl-2*	F: CTTTGTGGAGCTGTATGGGC	141	XM_021099593.1
	R: GCCCGTGGACTTCACTTATG		
*Bax*	F: GGCCCTTTTGCTTCAGGGTTT	269	XM_003127290.5
	R: GACACTCGCTCAACTTCTTGG		
*Caspase3*	F: ACTGTGGGATTGAGACGGAC	140	NM_214131.1
	R: GAACCAGGATCCGTCCTTTG		
*AQP-1*	F: ACCTGCTGGCGATTGACTAC	146	NM_214454.2
	R: GTCATAGATGAGCACGGCCA		
*AQP-5*	F: TCCATTGGCCTGTCTGTCAC	105	NM_001110424.1
	R: TCGATTCATGACGACTGCGG		
*PGC*	F: CCGTATCTGCCGTTCTGTGTC	102	XM_005665970.3
	R: AGCCTCACCTCCCAAAGTCCT		
*IL1R2*	F: GGTGTCAATGTGACATGGCG	100	NM_001243363.2
	R: CTGGCACAATCCAGAGAGCA		
*COMP*	F: TCCTACCGCTGGTTCCTACA	94	XM_003123527.3
	R: CAACATTGCTGTCTGCCACC		
*ACAN*	F: GGACTTGGATGTTGGCAGGA	140	NM_001164652.1
	R: AGTCAGGAAGGCCAGAGGAT		
*ALDH1A2*	F: TAGTCCCAGTGCTCCAAGTG	249	XM_021094606.1
	R: CTGGAAAGCGGAAGAGGAGA		
*ACSL6*	F: AGAGAGAGTGATCTGAGGTCTG	84	XM_021084743.1
	R: TCTAGCTCGGATCTGGTAGAGG		
*ACSM5*	F: GCCAGCTATGGACTGGGTTT	89	XM_003124563.6
	R: CCAGTGTCAGTCGTGTTCCA		
*AKR1C1*	F: TCCTCATAGGAGCAACAGGTG	127	XM_013980353.2
	R: CCAGAGCTTCACTCTTGGGAA		
*GNG4*	F: GGTTGTCAGGAAGCTGTGTG	181	XM_021072991.1
	R: CTTCAGCTTCTCGGATGTGC		
*GYS2*	F: GCAAAGCTGTGGACACGATG	119	NM_001195511.1
	R: GTCCAGGTTCCAAGCTGAGT		
*NR4A3*	F: CTCCTCCACTCTCAGCCTCT	83	NM_214247.1
	R: CACGCAGGGCATATCTGGAG		
*NR4A2*	F: AAGTCCCTTTCAAATGGCAGC	147	XM_005657531.3
	R: TCTTCTCAATCCCAGAGCCTT		
*MUC5B*	F: GCAACTACGTCTTCTCAGCG	115	XM_021082487.1
	R: TGGGTCTTGAGGACGATGTG		
*MUC5AC*	F: GTCAATGGCCGCACAATTCAG	129	XM_021082583.1
	R: CAAGAGACTGTCGTCCTGGTT		
*MMP9*	F: ACTCTTCACTCGGGACGGTA	147	NM_001038004.1
	R: TTGTCCTGGTCGTAGTTGGC		
*AMCF-II*	F: CGCTCTCTTCGCCACTATGA	133	NM_213876.1
	R: GATAGGACTAGCGCTGGCAA		
*IL22RA1*	F: AACATCCTGACGTGGGACAG	142	XM_021095535.1
	R: CCAAGGTCAGGTTGCAGGAT		
*CCL16*	F: CCTGCTGCCTGACGTATCAT	117	XM_003131714.4
	R: TCTCTCGGTTCCGTTTGGTG		
*SOX9*	F: GACTGCTGAATGAGAGCGAGA	122	NM_213843.2
	R: GTTCTTCACCGACTTTCTCCG		
*KRT5*	F: TCAGCACCTCCAGACCACTA	80	XM_003126173.4
	R: GACACACTCGATTGGCGAGA		
*GAPDH*	F: AGTATGATTCCACCCACGGC	139	NM_001206359.1
	R: TACGTAGCACCAGCATCACC		

## Data Availability

The data have been deposited in the NCBI BioProject database under accession number PRJNA1297621.

## SUPPLEMENTARY MATERIAL

**Supplement 1.** Data filtration statistics table.

**Supplement 2.** GO enrichment analysis results of differentially expressed genes in Hezuo pig. **Supplement 3.** KEGG enrichment analysis results of differentially expressed genes in Hezuo pig.

**Supplement 4.** KEGG enrichment analysis results of differentially expressed genes in Bama pig.
