# Supplementary material for: Distinct damage levels and transcriptional responses of lung in Hezuo pigs and Bama pigs during cold exposure
Source: Anim Biosci. 2026 Mar 11;39(6):250933. doi: 10.5713/ab.250933 (PMC13243925; doi:10.5713/ab.250933)
Supplement: Supplementary file 1 [file ab-250933-Supplementary-1.pdf]

Supplement 1. Data filtration statistics table

Sample	GC(%)	Q20(%)	Q30(%)	Clean Reads(%)	Clean Data(%)
BC1	47.99	98.32	95.19	98.73	98.49
BC2	48.15	98.32	95.17	98.7	98.45
BC3	48.12	98.37	95.28	98.8	98.55
BC4	47.67	98.37	95.29	98.81	98.51
BC5	47.83	98.28	95.04	98.29	98.01
BT1	48.1	98.4	95.41	98.81	98.54
BT2	47.92	98.37	95.32	98.77	98.5
BT3	48.09	98.35	95.22	98.84	98.59
BT4	47.84	98.34	95.22	98.79	98.53
BT5	48.25	98.33	95.19	98.77	98.5
HC1	47.55	98.37	95.28	98.82	98.59
HC2	47.74	98.38	95.32	98.78	98.52
HC3	47.67	98.4	95.34	98.77	98.51
HC4	47.68	98.4	95.4	98.76	98.49
HC5	47.86	98.44	95.48	98.79	98.51
HT1	47.76	98.33	95.17	98.79	98.54
HT2	48.31	98.39	95.32	98.77	98.54
HT3	47.91	98.39	95.33	98.82	98.59
HT4	47.83	98.45	95.5	98.86	98.62
HT5	47.72	98.38	95.28	98.77	98.52
