# Supplementary material for: Distinct damage levels and transcriptional responses of lung in Hezuo pigs and Bama pigs during cold exposure
Source: Anim Biosci. 2026 Mar 11;39(6):250933. doi: 10.5713/ab.250933 (PMC13243925; doi:10.5713/ab.250933)
Supplement: Supplementary file 2 [file ab-250933-Supplementary-2,3.pdf]

Supplement 2. GO enrichment analysis results of differentially expressed genes in Hezuo pig

Term	Pvalue	adjustPvalue	Up_gene	Down_gene
monocarboxylic acid metabolic process	2.55437e-08	8.46262e-05	VNN1; ACSL6; ALDH1A2; ACSM5; VNN2; etc.	AKR1C1; NR4A3; CYP2B22; CRABP2; CYP1A1); SLC27A2; etc.
ketosteroid monooxygenase activity	5.98661e-06	0.00243678	-	AKR1C1; etc.
response to corticotropin-releasing hormone	5.98661e-06	0.00243678	-	NR4A3; NR4A2; etc.
cellular response to corticotropin-releasing hormone stimulus	5.98661e-06	0.00243678	-	NR4A3; NR4A2; etc.

Supplement 3. KEGG enrichment analysis results of differentially expressed genes in Hezuo pig

Pathway	Pvalue	adjustPvalue	Up_gene	Down_gene	RichFactor
PI3K-Akt signaling pathway	0.00612719	0.119364	ALDH1A2; GYS2; VTN; LOC100038328; etc.	COMP; COL9A2; GNG4; NGFR; COL9A3; etc.	0.0302115
Retinol metabolism	5.87561e-06	0.00109874	ALDH1A2.	CYP2B22; LOC100515741; UGT2B31; etc.	0.118644
Arachidonic acid metabolism	0.00638311	0.119364	PLA2G5; PLA2G2F; etc.	AKR1C1; etc.	0.0645161
