# Supplementary material for: Distinct damage levels and transcriptional responses of lung in Hezuo pigs and Bama pigs during cold exposure
Source: Anim Biosci. 2026 Mar 11;39(6):250933. doi: 10.5713/ab.250933 (PMC13243925; doi:10.5713/ab.250933)
Supplement: Supplementary file 3 [file ab-250933-Supplementary-4.pdf]

Supplement 4. KEGG enrichment analysis results of differentially expressed genes in Bama pig

Pathway	Pvalue	adjustPvalue	Up_gene	Down_gene	RichFactor
Cytokine-cytokine receptor interaction	0.0202045	0.520326	NGFR; IL1R2; AMCF-II; IL22RA1; IL1RL1; CCL16	IL21; IL12B; CXCL11; etc.|	0.0426357
IL-17 signaling pathway	0.0377626	0.612293	MUC5AC; MUC5B; AMCF-II; SOX9	MMP9; MMP13	0.0568182
Viral protein interaction with cytokine and cytokine receptor	0.0317918	0.601395	AMCF-II; IL22RA1; CCL16	CCR7; CXCL11	0.0595238
